# Effects of temperature and heat waves on emergency department visits and emergency ambulance dispatches in Pudong New Area, China: a time series analysis

**DOI:** 10.1186/1476-069X-13-76

**Published:** 2014-10-02

**Authors:** Xiaoming Sun, Qiao Sun, Minjuan Yang, Xianfeng Zhou, Xiaopan Li, Aiqing Yu, Fuhai Geng, Yuming Guo

**Affiliations:** Health and Family Planning Commission of Pudong New Area, Shanghai, 200125 China; Center for Disease Prevention and Control of Pudong New Area, Shanghai, 3039 Zhangyang Rd, Shanghai, 200136 People’s Republic of China; Pudong New Area Weather Office, Shanghai, 200135 China; University of Queensland, Brisbane, Australia

**Keywords:** Temperature, Heat wave, Morbidity, Emergency department visits, Emergency ambulance dispatches

## Abstract

**Background:**

In July 2013, an extended heat episode with extreme high temperature covered Pudong New Area, the largest district in Shanghai. The current study estimates the impacts of temperature and heat waves on emergency department visits (EDV) and emergency ambulance dispatches (EAD) using time-series approaches in Pudong, from 2011 to 2013.

**Methods:**

An over-dispersed Poisson generalized additive model was used to examine the association between temperature and EDV and EAD. Heat wave effects with different heat wave definitions considering both the intensity and durations were also estimated.

**Results:**

Immediate effects of temperature on EDV and EAD were detected, after controlling for trends of time and day of week. The exposure-response relationships showed J-shaped curves with higher threshold temperature of EDV than that of EAD visually. When estimating risk changes on heat days compared with non-heat days using different percentiles of daily mean temperature in definition, EAD showed significant increases while non-significant or even negative associations were found for EDV. Heat wave with intensity above the 90th percentile had 2.62% (95% CI: 1.78%, 3.46%) and 0.95% (95% CI: 0.22%, 1.69%) increases in EDV for a duration of at least 2 days and 3 days respectively. The relative increase of EAD were 4.85% (95% CI: 1.42%, 8.39%) and 3.94% (95% CI: 0.88%, 7.10%).

**Conclusions:**

Varied effects of temperature and heat waves on emergency department visits and emergency ambulance dispatches were investigated. This wider view of the health effect of temperature indicated that interventions for both public health education and health services management should be considered in the study region.

**Electronic supplementary material:**

The online version of this article (doi:10.1186/1476-069X-13-76) contains supplementary material, which is available to authorized users.

## Background

As climate change received more global attention, more epidemiological studies have been conducted on the effects of temperature [1–4]. Recent epidemiologic evidence using modern statistical approaches, consisting primarily of the time-series and case-crossover approaches, mainly focused on the temperature-mortality relationship, including all-cause and cause-specific mortality, vulnerable subgroups, confounders and effect modifiers. Peng et al. concluded that the heat associated mortality and morbidity were expected to increase in a warming climate, particularly within the vulnerable populations of the elderly, children, those with chronic diseases, and people with special socio-economic status [5]. Anderson et al. found heat-related mortality was most associated with a shorter lag (average of same day and previous day). Acclimatization, individual susceptibility, and community characteristics all affect heat-related effects on mortality [6]. Basu et al. Characterized temperature and mortality in 9 california counties, no air pollutant examined was found to be a significant confounder or effect modifier [7]. Only a few studies have examined the impacts of temperature on morbidity, mainly concentrated on hospital admissions or heat-related emergency data [8–12]. Relevant literature before 2005 found contrasting patterns of hospital admission and mortality during heat waves, which could be explained by different sources of variation(country, data source, data quality) [8]. The 2006 California heat wave had a substantial effect on emergency department visits across the state [RR = 6.30; 95% (CI), 5.67–7.01], children (0–4 years of age) and the elderly (≥65 years of age) were at greatest risk [10]. A comparative study in Phoenix, Arizona and Chicago, Illinois using heat-related emergency calls data identified different threshold temperatures (35.0 in Chicago and 45.0 in Phoenix) and differences in the vulnerability and sensitivity of the cities. The study empirically demonstrated the need for improved adaptive capacity and suggested future strategies for achieving this goal [12].

A variety of temperature metrics have been applied across studies, including the ambient temperature (mean, maximum, minimum daily temperature), apparent temperature, diurnal temperature range, and extreme temperatures (heat waves) [13, 14]. The definition of heat wave varied across different study designs, in terms of intensity and duration of the heat. Some studies addressed the impacts of heat episodes [15, 16], while some examined the effect modification by different heat characteristics [17, 18], and some examined the heat slopes and identified threshold temperatures [19, 20]. The threshold temperature above which mortality increases, also called the minimum mortality temperature, can be an indicator of adaptive or acclimatization.

The current evidence suggested that the temperature impacts varied by geographic, climate, and population characteristics, but mainly from western developed countries [21–23]. Only few studies conducted in Asia developing countries like China, most of which unfortunately focused on associations between temperature or heat waves and mortality [24–26].

In July 2013, an extended heat with extreme high temperature covered Pudong New Area, the largest district in Shanghai. This unusual heat episode had broken the heat records of Shanghai in over 141 years, and underwent 16 days with a daily maximum temperature more than 38°C. The current study estimates the impacts of temperature and heat waves on emergency department visits and emergency ambulance dispatches using time-series approaches in Pudong, from 2011 to 2013. To our knowledge, this is the first study conducted in this region, so that specific public health policies can be implemented for this district rather than for the entire geographic area.

## Methods

### Study population

The current study was conducted in Pudong New Area (latitude 31.221 and longitude 121.544), the largest district in Shanghai, from 1 Jan 2011 to 31 Aug 2013. Pudong had a population of 5.26 million,within a land area of 1430 square kilometers, both representing 22% of Shanghai’ total. It has a maritime climate with four distinct seasons, the annual average temperature was 16.7°C, and the annual precipitation was 1343.9 mm (2012 Statistical Yearbook).

### Data

Emergency department visits (EDV) data were obtained from the Pudong Institute for Health Development. Patient visits in emergency department were recorded by the Health Information Systerm (HIS) of hospitals, a total number of all emergency department visits was computed everyday.

Emergency ambulance dispatches (EAD) data were available from Pudong Medical Emergency Center, the unique authority for pre-hospital medical service in Pudong. Each dispatch was recorded by the 120 Network Platform which connected with Global Position System (GPS). Each record contains the address of the dispatch destination, the day and time when the call was made. Although most of the records contains a complaint of the patient, they were in very general terms and were difficult to code using International Classification of Diseases (ICD). In this study only daily counts of all dispatches were used.

Meteorological data came from the Pudong New Area Weather Office, including mean, maximum, and minimum temperature, average relative humidity, average wind velocity, rainfall and atmospheric pressure. All weather data were delivered as daily measurements from the Century Park Automatic Monitoring Station (see detailed location in Additional file 1: Figure S1).

### Statistical analysis

We examined the temperature effect on EDV and EAD using an over-dispersed Poisson generalized additive model (GAM). This approach was originally developed and widely accepted in air pollution health effects studies [27, 28]. Long-term trends and seasonal patterns, as well as day of the week (DOW) were controlled in the first step. Possible effects of relative humidity and other weather variables were considered, but no significant information was found. The basic model structure is
1

where E(Y_*t*_) is the expected number of EDV and EAD, assumed to follow an over dispersed Poisson distribution on day *t*; β_0_ is the model intercept; s(Time_*t*_) is the regression spline function of a variable representing time to adjust for long-term trends and seasonal patterns, degrees of freedom (DF) for trend was selected automatically by the Generalized Cross Validation (GCV) criterion; DOW_*t*_ is the categorical variable for day of the week; s(T_*t*_) is the spline function of temperature on day *t*, it should be *a*(T_*t*_) when treating temperature as a linear term and *a* is the vector of effect coefficients.

Lag structures were considered to capture any delayed effects of temperature, with the temperature term on the same day and also moving average values up to 7 days before.

To examining the effects of high temperature and heat waves, only data during the warm season (May-September, 429 days) were analyzed, with an attempt to exclude possible effects from low temperatures (like cold-related health effects). A term of heat (heat wave) was introduced into the basic model structure (equation 1), and the new model structure is
2

where s(DOY_*t*_) is a spline function for day of the year to describe the seasonal pattern [29]; Year_*t*_ is a categorical variable of calendar year for 2011–2013; *a* is the vector of regression coefficients for EDV or EAD on heat (heat wave) days versus non-heat (non-heat wave) days; HW_*t*_ is 0 if day *t* is a non-heat (non-heat wave)day, 1 if day *t* is a heat (heat wave) day.

Different definitions of heat waves were used consisting both of the intensity and duration:

Heat (single day): days with daily mean temperature above 90th (29.67°C), 95th (30.70°C) and 99th (33.05°C) percentiles of the whole years, respectively;

Heat waves: a period of heat days with duration of at least 2 or 3 days.

The data were analyzed using the package “mgcv” version 1.7-22 in the statistical software R version 3.0.1 (2013 The R Foundation for Statistical Computing) [30]. All statistical analyses were two-sided and significance was set at *P* < 0.05.

### Sensitivity analyses

We applied several sensitivity analyses. First, we considered other temperature definitions. Different metrics of temperature (the minimum and maximum temperature rather than the mean temperature) were introduced into the basic model separately. We also examined robustness of the estimates through incorporating a range of DFs for temporal spline, by using fixed DFs for the regression spline function. Third, we removed the spikes in the EDV data by removing the upper 95% daily EDV values of an individual year separately, and re-run our analyses to see if the results were largely influenced by these unknown events.

## Results

A total of 4,450,556 EDV and 246,372 EAD were enrolled during the study period. Table 1 summarizes the morbidity indicators and meteorological variables in Pudong, Jan 2011-Aug 2013 (974 days). Daily counts of EDV and EAD were 4579 and 253 respectively. During the study period, the mean, maximal and minimal temperatures were 17.2°C, 21.3°C and 13.9°C.

**Figure 1:**
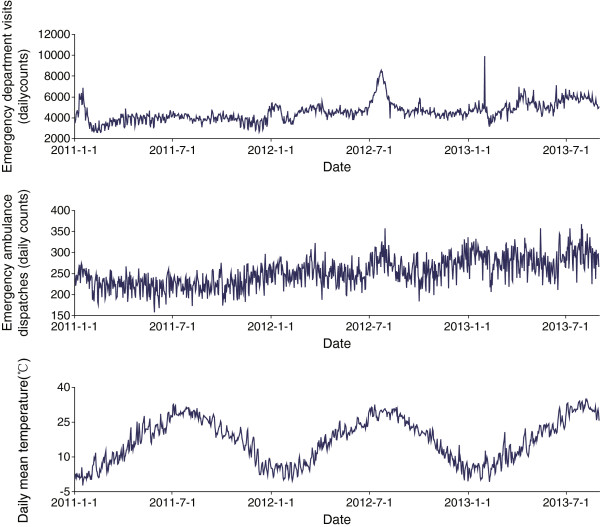
**Emergency department visits (upper), emergency ambulance dispatches (middle) and daily mean temperature (down) in Pudong, 2011–2013.**

Figure 1 shows the time series of EDV and EAD, along with the daily mean temperature. No obvious seasonal patterns showed in these two morbidity indicators. The EDV data showed three spikes: one early in 2011, another in July 2012, and a third on Jan. 30th 2013. As no obvious cause was detected after considering holidays and influenza information, we re-run the analyses by removing this three spikes in our sensitivity analyses (results shown later).

**Table 1:** **Characteristic statistics of emergency department visits and emergency ambulance dispatches and meteorological variables in Pudong, Shanghai, during 2011-2013**

Variables	Mean	SD	Min	Percentiles			Max
				25	50	75	
Emergency department visits	4579	920	2578	3983	4472	5020	9862
Emergency ambulance dispatches	253	35	159	228	251	276	367
Mean temperature (°C)	17.2	9.4	−2.2	8.4	18.6	24.8	34.8
Maximal temperature (°C)	21.3	9.8	0.6	12.4	22.5	29.3	40.9
Minimal temperature (°C)	13.9	9.5	−5.0	5.4	14.8	22.4	29.3
Relative humidity (%)	71	12	31	63	73	80	97
Wind velocity (m/s)	1.7	0.7	0.1	1.2	1.6	2.1	6.1

### Effect estimates of daily mean temperature

**Figure 2:**
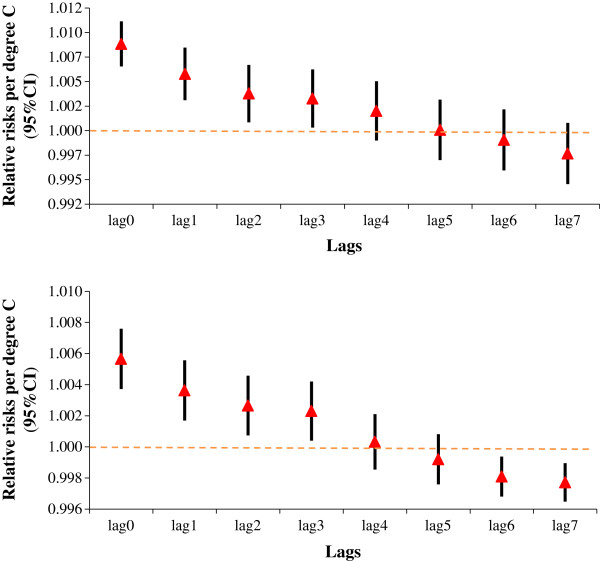
**Relative risks of daily mean temperature on emergency department visits and emergency ambulance dispatches with different lags in Pudong, 2011–2013.** The triangles reflect the central estimates of relative risks per degree C and the vertical lines show the 95% confidence intervals. Model controlled for trends of time and day of week.

Figure 2 shows the effects of daily mean temperature on EDV and EAD along the lags up to 7 days respectively. Model controlled for trends of time and day of week. Temperature showed immediate effects on both of the morbidity indicators, with greatest effects on the same day as event (lag0).

**Figure 3:**
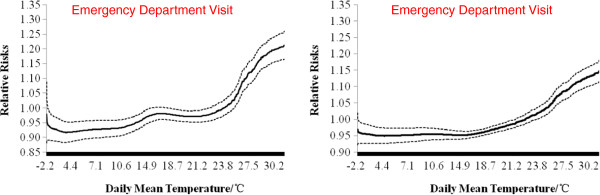
**General relationship between daily mean temperature and emergency department visits (left), emergency ambulance dispatches (right) in Pudong, 2011–2013.** The dotted line show the 95% confidence intervals. Model controlled for trends of time (df = 9), and day of week.

Figure 3 shows the exposure-response relationship between daily mean temperature and EDV and EAD at the same day as event (lag0), after controlling for confounders in the models (plots of the time term in statistical model equation 1 were provided in Additional file 1: Figure S2). Both of the relationship showed J-shaped curves, indicating a one-threshold model of the temperature-morbidity association. The threshold temperature (which indicates that morbidity level will increase if the temperature increases from this point) of EDV is higher than that of EAD visually. The increase in relative risk for per unit increase of temperature is also higher for EDV as shown by the steeper slope on the right-hand side of the curve. However, the estimated spline curve of EDV decreased when daily mean temperature exceeded around 30°C.

### Effect estimates of high temperature and heat waves

Table 2 shows the risks of high temperature on EDV and EAD after controlling for day of the year, day of week and calendar year, using data only from the warm season (May-September). Every 1°C increase in daily mean temperature showed a 0.60% increase (95% Confidence interval (CI): 0.53%, 0.66%) in EDV, and a 1.25% increase (95% CI: 0.97%, 1.53%) in EAD (plots of the time term in statistical model equation 2 were provided in Additional file 1: Figure S3). When estimating risk changes on heat days compared with non-heat days using different percentiles of daily mean temperature in definition, EAD showed significant increases while non-significant or even negative associations were found for EDV.

**Table 2:** **Percent changes of risks on emergency department visits and emergency ambulance dispatches for high temperature in Pudong, May-September (2011–2013)**

Linear variables	Percent changes (%) (95% CI)	
	Emergency department visits	Emergency ambulance dispatches
Temperature^a^	0.60(0.53 ~ 0.66)	1.25(0.97 ~ 1.53)
Heat90^b,c^	0.92(−0.08 ~ 1.93)	6.03(2.03 ~ 10.18)
Heat95^b,c^	−4.06(−4.97 ~ −3.14)	−1.48(−5.33 ~ 2.52)
Heat99^b,c^	−6.51(−8.40 ~ −4.59)	8.79(0.37 ~ 17.92)

^a^model controlled for day of the year, day of week and calendar year; daily mean temperature applied as a linear term.

^b^definitions of heat (heat90, heat95, heat99): days at temperatures above the 90th (29.67°C), 95th (30.70°C) and 99th (33.05°C) percentile daily mean temperature respectively.

^c^Risks on heat days compared with non-heat days; model controlled for day of the year, day of week and calendar year; daily mean temperature included into the model as a spline term.

Table 3 shows percent changes of risks on heat wave days compared with non-heat wave days for EDV and EAD under different definitions of heat wave. The results indicated strong associations between heat waves and morbidity outcomes, although the risks varied depend on different definitions used. For instance, heat wave with intensity above the 90th percentile, had 2.62% (95% CI: 1.78%, 3.46%) and 0.95% (95% CI: 0.22%, 1.69%) increases in EDV for a duration of at least 2 days and 3 days respectively. The relative increase of EAD were 4.85% (95% CI: 1.42%, 8.39%) and 3.94% (95% CI: 0.88%, 7.10%). Negative or non-significant results were found for heat waves with higher intensity, except for the heat waves with intensity above 99th percentile.

**Table 3:** **Heat wave risks on emergency department visits and emergency ambulance dispatches under different definitions in Pudong, May-September (2011–2013)** ^**a**^

Medical emergency activities	Intensity	≥2 days duration		≥3 days duration	
		No. of heat waves (days)	Percent changes (%) (95% CI)	No. of heat waves (days)	Percent changes (%) (95% CI)
Emergency department visits	≥90th percentile	17 (93)	2.62 (1.78 ~ 3.46)	15 (89)	0.95 (0.22 ~ 1.69)
	≥95th percentile	11 (44)	−1.73 (−2.59 ~ −0.87)	5 (32)	−9.67 (−10.49 ~ −8.84)
	≥99th percentile	3 (9)	−10.21(−12.10 ~ −8.29)	1 (5)	12.46 (10.33 ~ 14.62)
Emergency ambulance dispatches	≥90th percentile	17 (93)	4.85 (1.42 ~ 8.39)	15 (89)	3.94 (0.88 ~ 7.10)
	≥95th percentile	11 (44)	−2.48 (−6.04 ~ 1.22)	5 (32)	−2.69 (−6.40 ~ 1.16)
	≥99th percentile	3 (9)	10.22 (1.44 ~ 19.76)	1 (5)	3.02 (−4.68 ~ 11.35)

^a^model controlled for day of the year, day of week, calendar year and temperature as a spline term. Percent changes (95% CI) in estimated risks on heat-wave days compared with non-heat-wave days were presented. Daily mean temperature of percentiles were 90th (29.67°C), 95th (30.70°C) and 99th (33.05°C), respectively.

### Sensitivity analyses

The sensitivity analyses using different temperature metrics in equation 1 yielded similar results, the effects on EDV and EAD decreased a little when using daily maximum and minimum temperature respectively (results showed in Additional file 1: Table S1 and Additional file 1: Figure S4). To examining the robustness of the estimates, our sensitivity analyses incorporated a range of DF for temporal spline. For the same day mean temperature, when the DF of time changed from 9–17, the effects on EDV fluctuated between 0.84%/°C and 1.14%/°C, which were close to the estimate (0.83%/°C) when DF of time was selected automatically by the GCV criterion. Similarly, there were minor changes for EAD (results not shown).

We re-run our analyses by removing the spikes in the EDV data, similar estimates were observed. Every 1°C increase in daily mean temperature corresponded to a 0.90% increase (95% CI: 0.67%, 1.12%) of EDV (results showed in Additional file 1: Table S2).

## Discussion

In this study, we analyzed the association between temperature and two kinds of morbidity indicators, EDV and EAD. Immediate effects of temperature were found, both with a greatest effect on the same day as event (lag0). Similar exposure-response relationships were found for these two temperature-morbidity associations, basically in ‘J’-shaped version. The estimated spline curve of EDV showed a higher threshold temperature and may reflect the lower level of heat sensitivity. But the slope decreased when daily mean temperature exceeded around 30°C visually which may indicating acclimatization and self-protection of the population, as some of the emergency department visits were not very urgent cases, and patients without severe illnesses may choose to stay at home and delay their hospital visit activity on days with extreme temperature. We also estimated heat wave effects with different definitions considering both the intensity and durations, using data of the warm season (May-September). Heat wave with intensity above the 90th percentile and a duration of at least 2 days or 3 days, had 2.62% (95%CI: 1.78%, 3.46%) and 0.95% (95% CI: 0.22%, 1.69%) increases in EDV, 4.85% (95% CI: 1.42%, 8.39%) and 3.94% (95% CI: 0.88%, 7.10%) increases in EAD respectively.

While the temperature-mortality relationship was widely investigated, less attention was paid into research of morbidity indicators. Study conducted in Brisbane, Australia found significant association between temperature and emergency admissions for stroke, its impact varied with different type of stroke [31]. Green et al. found that a 10 degrees F increase in mean apparent temperature was associated with a 3.5% (95% CI: 1.5, 5.6) increase in ischemic stroke and increases in several other disease-specific outcomes in nine California counties [32]. Strong effects were showed with diabetic and circulatory visits per 1°C increase in temperature above an identified threshold in Chiang Mai, Thailand [33].

Consistent with the evidence found with temperature, increased hospitalizations have been associated with heat waves in Chicago [34], Australia [11], California [10] and Canada [35]. Although no standard definition of heat waves were used, most studies applied a combination of intensity and duration [17, 18, 21, 36]. Some studies also suggested heat waves in early summer seemed to cause greater health effects [37]. A population-based cohort study in Taiwan identified the annual first extreme heat event with the temperature of 99th percentile was associated with elevated ERV for all cause and cardiopulmonary diseases [38].

Given the lack of research into temperature and ambulance dispatches, the current study contributes to an understanding of the impact of temperature and heat waves on morbidity indicators in China. In previous studies, a Toronto study reported large increases in ambulance response calls during the summer period [39], studies in Italy and Australia found immediate and short-term increasing risks of heat on ambulance dispatches [40, 41]. A case-series study in the Adelaide metropolitan population found a 4% (95% CI: 1%, 7%) increase of total ambulance transport during heat waves [42]. As the ambulance dispatch data is an important resource in public health activities and is effectively available in real-time, while the mortality data can only be considered reliable after several days, the use of emergency ambulance dispatches as an indicator of health effects of heat episodes other than mortality is efficient.

The current study has some limitations. First, the study was limited to a relatively short period, the conclusions comparisons with other studies should be made with caution. Studies to identify the vulnerable subgroups of mortality from high ambient temperature found differences in susceptibility relating to socioeconomic factors [9, 32, 43]. Further investigation using individual-level data is needed. The possible confounding by air pollution was not considered in this study as the data was not available. In previous studies considering the effects of temperature on mortality, a high correlation was found between temperature and air pollutants, but some found no effect modification or confounding by pollution [7, 44].

However, this is the first study to our knowledge exploring the heat wave effects on non-fatal health outcomes using two types of morbidity indicators, the emergency department visits and emergency ambulance dispatches in China. One of the key contributions of this study is improving understanding of the heat-morbidity relationship in Shanghai, China. As emergency is considered to be less severe and more acute, and can catch the effect of temperature at the early stage even pre-hospital conditions, also the ambulance dispatches are effectively available in real-time, the current heat warning system which was typically implemented based on mortality must take into account heat-related morbidity indicators. Our study achieved a wider view of the health effect of temperature and heat episodes in the study region, interventions for both public health education and health services management should be considered. First, it can inform the municipal government to improve their public health education plans about the hazards of heat; the immediate effects of temperature suggests more accurate weather forecast as this is the most important input; an appropriate response to heat alerts, especially for pre-hospital health services and clinical resource allocation is also needed.

## Conclusions

The current study identified the heat-morbidity relationship in Pudong, China. Varied effects of temperature and heat waves on emergency department visits and emergency ambulance dispatches were detected. This study achieved a wider view of the health effect of temperature in the study region, interventions for both public health education and health services management should be considered.

## Electronic supplementary material

Additional file 1: **This file contains two tables and four figures to the manuscript.** **Table S1.** Percent changes of risks on emergency department visits and emergency ambulance dispatches using different temperature metrics, Pudong (2011–2013). Model controlled for trends of time and day of week. **Table S2.** Percent changes of relative risks on emergency department visits in sensitivity analyses, Pudong (2011–2013). Model controlled for trends of time and day of week. The upper 95% daily EDV values of an individual year were removed separately. **Figure S1.** Pudong New Area of Shanghai (black area) and Century Park Automatic Monitoring Station (red square). **Figure S2.** Plots of time term in the basic statistical model of emergency department visits data (Left) and emergency ambulance dispatches data (Right). The solid lines reflect the estimated relative risks, the dotted lines show the 95% confidence intervals. Model controlled for trends of time and day of week. **Figure S3.** Plots of time term in the statistical model (warm season analysis) of emergency department visits data (Left) and emergency ambulance dispatches data (Right). The solid lines reflect the estimated relative risks, the dotted lines show the 95% confidence intervals. Model controlled for day of the year, day of week and calendar year. **Figure S4.** General relationship between different temperature terms (daily mean temperature, daily maximum temperature, daily minimum temperature) and emergency department visits (Left), emergency ambulance dispatches (Right) in Pudong, 2011–2013. The dotted lines show the 95% confidence intervals. Model controlled for trends of time and day of week. (DOCX 716 KB)

## Abbreviations

EDV: Emergency department visits
EAD: Emergency ambulance dispatches
GAM: Generalized additive model
DOW: Day of the week
GCV: Generalized cross validation
DF: Degrees of freedom
DOY: Day of the year
CI: Confidence interval.
