# Supplementary material for: Effects of temperature and heat waves on emergency department visits and emergency ambulance dispatches in Pudong New Area, China: a time series analysis
Source: Environ Health. 2014 Oct 2;13:76. doi: 10.1186/1476-069X-13-76 (PMC4201734; doi:10.1186/1476-069X-13-76)

**Additional file 1 Table S1. Percent changes of risks on emergency department visits and emergency ambulance dispatches using**

**different temperature metrics, Pudong (2011-2013).**

| Temperature Variables^a^ | Percent changes(%)(95%CI) | |
| --- | --- | --- |
|  | Emergency department visits | Emergency ambulance dispatches |
| Daily Mean Temperature | 0.83（0.61~1.06） | 0.57（0.37~0.76） |
| Daily Maximum Temperature | 0.68（0.48~0.87） | 0.41（0.27~0.56） |
| Daily Minimum Temperature | 0.58（0.33~0.83） | 0.34（0.15~0.53） |

a: model controlled for trends of time and day of week.

**Additional file 1 Table S2. Percent changes of relative risks on emergency department visits in sensitivity analyses, Pudong (2011-2013)** ^a,b^**.**

| Temperature Variables | Relative Risks Percent changes(%)(95%CI) |
| --- | --- |
| Daily Mean Temperature | 0.90（0.67~1.12） |
| Daily Maximum Temperature | 0.73（0.56~0.90） |
| Daily Minimum Temperature | 0.45（0.23~0.66） |

a: model controlled for trends of time and day of week.

b: upper 95% daily EDV values of an individual year were removed separately.

**Additional file 1 Figure S1. Pudong New Area of Shanghai (black area) and Century Park Automatic Monitoring Station (red square).**

**Shang Hai**

**Pudong New Area**

**Zhe Jiang**

**Jiang Su**

**Additional file 1 Figure S2. Plots of time term in the basic statistical model of emergency department visits data (Left) and emergency ambulance dispatches data (Right). The solid lines reflect the estimated relative risks, the dotted lines show the 95% confidence intervals. Model controlled for trends of time and day of week.**


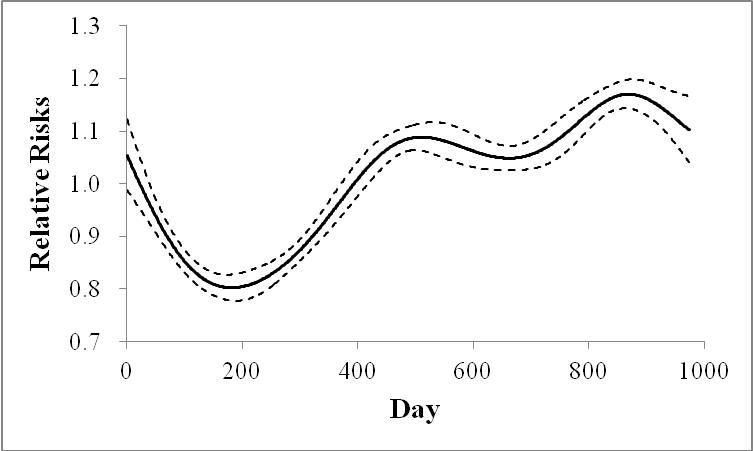

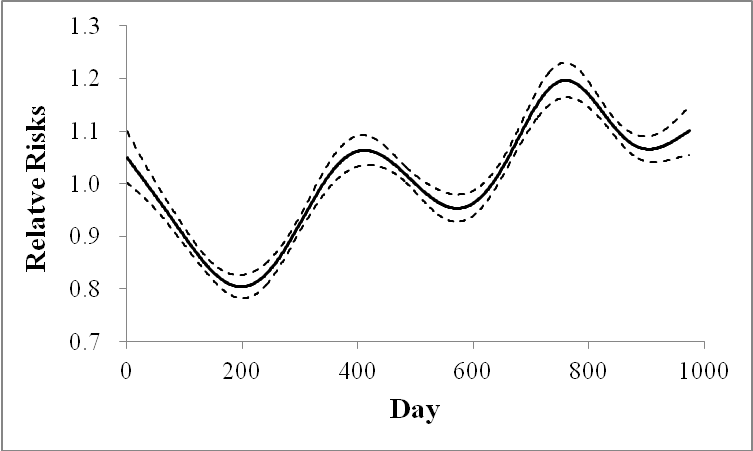


**Additional file 1 Figure S3. Plots of time term in the statistical model (warm season analysis) of emergency department visits data (Left) and emergency ambulance dispatches data (Right). The solid lines reflect the estimated relative risks, the dotted lines show the 95% confidence intervals. Model controlled for day of year, day of week and calendar year.**


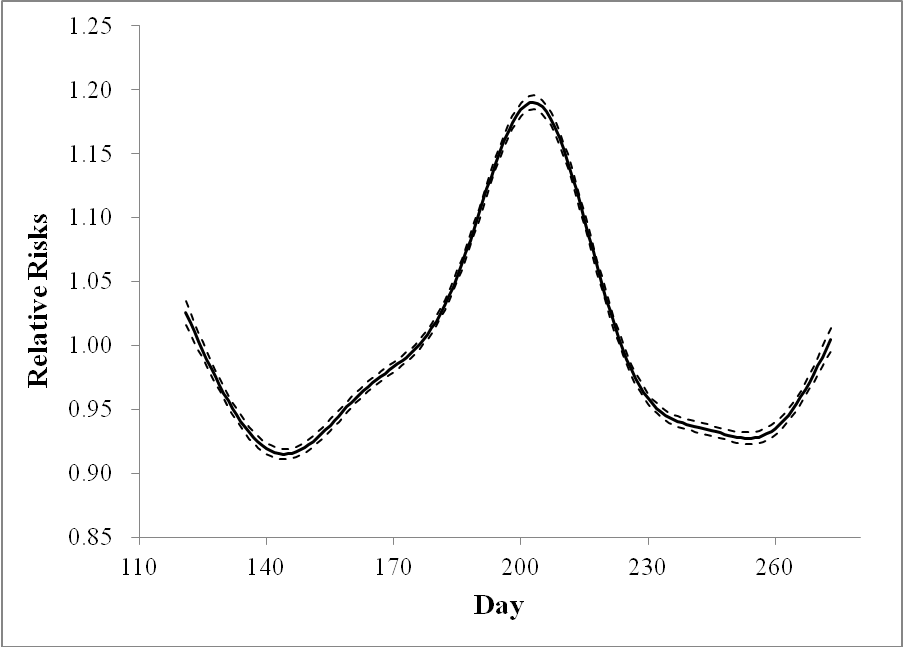

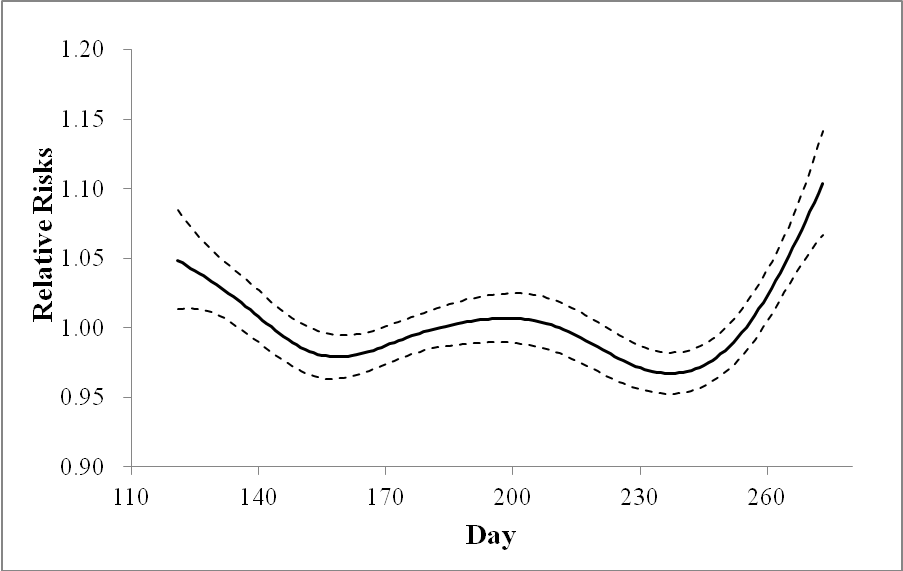


**Additional file 1 Figure S4. General relationship between different temperature terms (daily mean temperature, daily maximum temperature, daily minimum temperature) and emergency department visits (Left), emergency ambulance dispatches (Right) in Pudong, 2011-2013. The dotted line show the 95% confidence intervals. Model controlled for trends of time and day of week.**


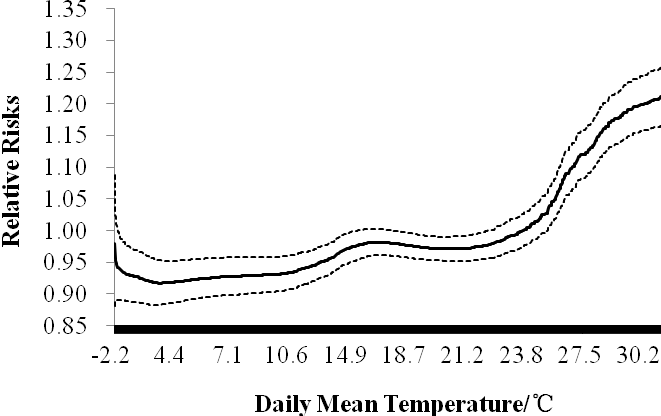

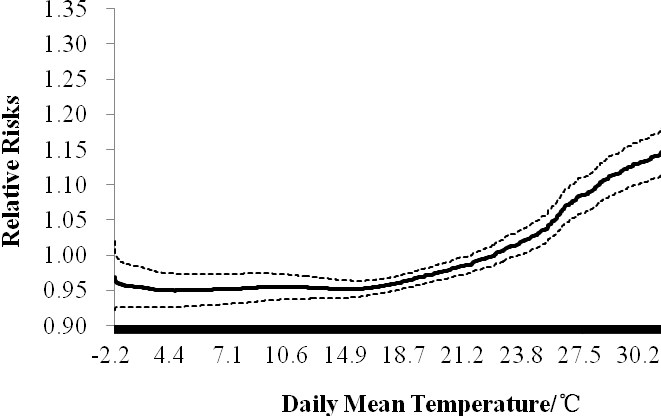

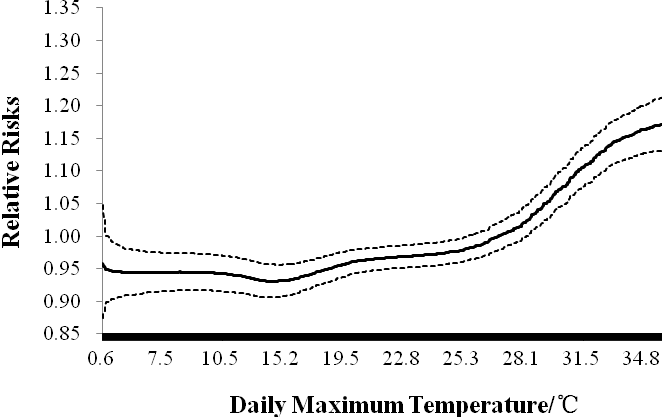

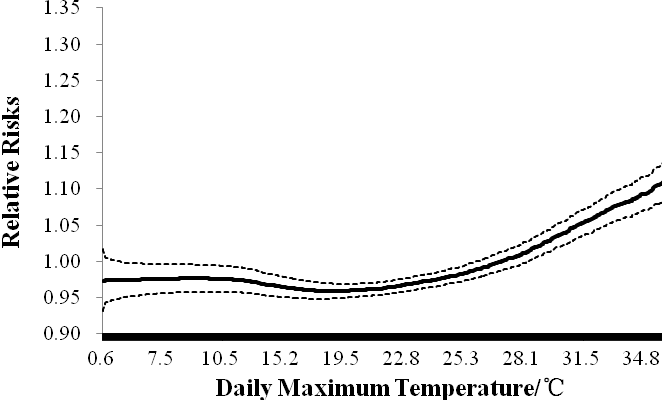

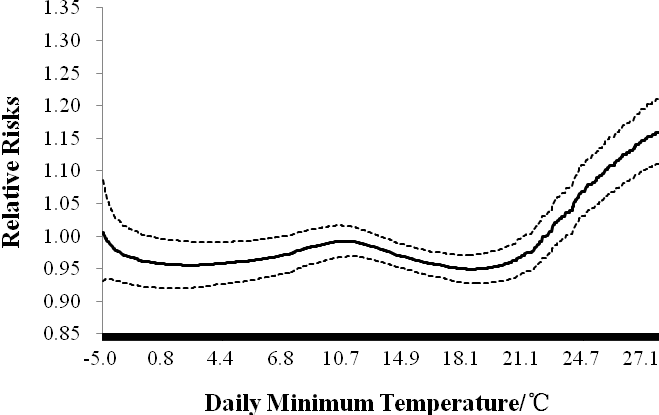

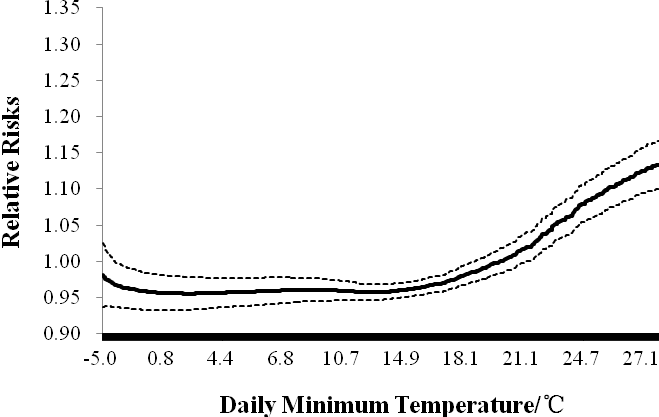

Supplement: Supplementary file 1 — Additional file 1: This file contains two tables and four figures to the manuscript. Table S1. Percent changes of risks on emergency department visits and emergency ambulance dispatches using different temperature metrics, Pudong (2011–2013). Model controlled for trends of time and day of week. Table S2. Percent changes of relative risks on emergency department visits in sensitivity analyses, Pudong (2011–2013). Model controlled for trends of time and day of week. The upper 95% daily EDV values of an individual year were removed separately. Figure S1. Pudong New Area of Shanghai (black area) and Century Park Automatic Monitoring Station (red square). Figure S2. Plots of time term in the basic statistical model of emergency department visits data (Left) and emergency ambulance dispatches data (Right). The solid lines reflect the estimated relative risks, the dotted lines show the 95% confidence intervals. Model controlled for trends of time and day of week. Figure S3. Plots of time term in the statistical model (warm season analysis) of emergency department visits data (Left) and emergency ambulance dispatches data (Right). The solid lines reflect the estimated relative risks, the dotted lines show the 95% confidence intervals. Model controlled for day of the year, day of week and calendar year. Figure S4. General relationship between different temperature terms (daily mean temperature, daily maximum temperature, daily minimum temperature) and emergency department visits (Left), emergency ambulance dispatches (Right) in Pudong, 2011–2013. The dotted lines show the 95% confidence intervals. Model controlled for trends of time and day of week. (DOCX 716 KB) [file 12940_2013_785_MOESM1_ESM.docx]
